# Essential Oil-Loaded NLC for Potential Intranasal Administration

**DOI:** 10.3390/pharmaceutics13081166

**Published:** 2021-07-28

**Authors:** Angela Bonaccorso, Cinzia Cimino, Daniela Erminia Manno, Barbara Tomasello, Antonio Serra, Teresa Musumeci, Giovanni Puglisi, Rosario Pignatello, Claudia Carbone

**Affiliations:** 1 Laboratory of Drug Delivery Technology, Department of Drug and Health Sciences, University of Catania, Viale A. Doria 6, 95124 Catania, Italy; angela.bonaccorso@unict.it (A.B.); cinzia.cimino@phd.unict.it (C.C.); barbara.tomasello@unict.it (B.T.); teresa.musumeci@unict.it (T.M.); puglisig@unict.it (G.P.); rosario.pignatello@unict.it (R.P.); 2 Dipartimento di Matematica e Fisica, University of Salento, 73100 Lecce, Italy; daniela.manno@unisalento.it (D.E.M.); antonio.serra@unisalento.it (A.S.)

**Keywords:** nanomedicine, nose-to-brain, *Lavandula*, *Mentha*, *Rosmarinus*, TEM, cell viability, mucoadhesion

## Abstract

Complementary and alternative medicines represent an interesting field of research on which worldwide academics are focusing many efforts. In particular, the possibility to exploit pharmaceutical technology strategies, such as the nanoencapsulation, for the delivery of essential oils is emerging as a promising strategy not only in Italy but also all over the world. The aim of this work was the development of nanostructured lipid carriers (NLC) for the delivery of essential oils (*Lavandula*, *Mentha*, and *Rosmarinus*) by intranasal administration, an interesting topic in which Italian contributions have recently increased. Essential oil-loaded NLC, projected as a possible add-on strategy in the treatment of neurodegenerative diseases, were characterized in comparison to control formulations prepared with Tegosoft CT and Neem oil. Homogeneous (polydispersity index, PDI < 0.2) nanoparticles with a small size (<200 nm) and good stability were obtained. Morphological and physical-chemical studies showed the formation of different structures depending on the nature of the liquid oil component. In particular, NLC prepared with *Lavandula* or *Rosmarinus* showed the formation of a more ordered structure with higher cytocompatibility on two cell lines, murine and human fibroblasts. Taken together, our preliminary results show that optimized positively charged NLC containing *Lavandula* or *Rosmarinus* can be proposed as a potential add-on strategy in the treatment of neurodegenerative diseases through intranasal administration, due to the well-known beneficial effects of essential oils and the mucoadhesive properties of NLC.

## 1. Introduction

Essential oils (EOs) are important natural mixtures produced by aromatic plants during their secondary metabolism, characterized by the presence of monoterpenes and sesquiterpenes, with other important aliphatic compounds, including terpenoids, alcohols, ethers, esters, ketones, and aldehydes [1]. Their quali-quantitative composition is influenced by several factors, including the variety of the plant, growing place (in particular, environment, climate, and eventual stress suffered), nutrition and fertilizers used, and extraction method. Due to the many properties of pharmaceutical interest (antioxidant, anti-inflammatory, antimicrobial, wound-healing, and anxiolytic), an increasing interest in complementary and alternative medicines (CAMs) has spread during the last years, with several studies exploring the potential use of EOs as adjuvants in various diseases, in particular when encapsulated into vesicular or nanoparticulate delivery systems [1,2,3,4,5,6,7,8,9,10,11,12,13,14,15,16]. The advantages of EOs encapsulation are related to the possibility to overcome different drawbacks by enhancing their stability, providing a controlled release and thus increasing their bioavailability and effectiveness.

As recently described by Scuteri et al. [12], the use of EOs via inhalation (aromatherapy), such as *Lavandula officinalis* and *Melissa officinalis*, represents a complementary approach in the treatment of Alzheimer’s disease (AD), since the treatment with antipsychotic drugs is limited by the short-term use (maximum 12 weeks). Even if the mechanisms of actions of EOs are still not clear, their ability to bind to the olfactory nerve system is responsible for the transmission of the signal to specific areas of the central nervous systems (hippocampus, limbic system, amygdala, and hypothalamus). The potential application of different EOs in AD treatment was also reported by Jimbo et al. [17], who developed a clinical trial on 28 elderly people affected by dementia, demonstrating the importance of aromatherapy in AD treatment. The success of the treatment, demonstrated by the resulting significant improvement in cognitive function, was based on the use of a combined therapy, with two administrations per day for 28 days, of four different EOs. In the morning, 0.04 mL of *Citrus limon* (L.) and 0.08 mL of *Rosmarinus officinalis* were administered based on the idea that this mixture is able to activate the sympathetic nervous system, thus promoting concentration and memory. In the evening, aiming to induce relaxation through the activation of the parasympathetic nervous system, a mixture constituted of 0.04 mL of *Citrus sinensis* (L.) Osbeck and 0.04 mL *Lavandula angustifolia* was administered. Interestingly, Rinaldi et al. recently developed chitosan coated nanoemulsions for the intranasal delivery of *Thymus vulgaris* and *Syzygium aromaticum* EOs, in the treatment of brain infections (meningitis and encephalitis) caused by bacterial strains of clinical concern [18]. The good mucoadhesive properties of nanoemulsions would enhance EOs’ nasal administration, thus allowing EOs’ antibacterial activity, as demonstrated on different bacterial strains [18].

Based on these considerations, the aim of this work was the development of EO-loaded nanostructured lipid carriers (NLC) for intranasal administration as potential adjuvant in the treatment of neurodegenerative diseases. Polymeric and lipid nanoparticles, solid lipid nanoparticles (SLN), and second-generation NLC have been widely investigated for brain targeting [18,19,20,21,22,23,24,25,26,27,28,29,30,31]. Herein, NLC were selected for their ability to directly release active compounds to the brain endothelial cells, as recently demonstrated by Arduino et al. [32]. *Rosmarinus officinalis* L., *Lavandula* × *intermedia* “Sumian”, and *Mentha piperita* were selected due to their potential beneficial effects reported in AD treatment. *Lavandula* [33] and *Rosmarinus* [34] showed promising results in behavioral tests, demonstrating their ability to provide benefits in the treatment of scopolamine-induced Alzheimer’s-type dementia. *Lavandula* was able to reduce depression, anxiety, and memory impairment [33], while *Rosmarinus* increased imaged and number memory in a trial carried out on school students [35]. *Mentha* EO was also found to enhance memorization process, through its ability to inhibit both acetylcholinesterase (AChE) and butyrylcholinesterase (BuChE) in a dose-dependent manner, thus suggesting a potential use in the treatment of neurodegenerative disorders [36].

EO-loaded NLC were characterized from a physical-chemical and technological point of view in comparison to NLC prepared with Tegosoft CT oil, used as EO-free control formulation, and NLC loaded with Neem oil, used as negative control due to its documented toxic effects [37,38,39]. All NLC formulations were analyzed to determine their mean size, polydispersity, and zeta potential by photon correlation spectroscopy (PCS) analysis. NLC were also characterized in terms of osmolarity, pH measurements, entrapment efficiency (EE%) of the loaded EO, and stability over time exploiting Turbiscan^®^ AG Station. In order to highlight the potential influence of the liquid oil component on NLC structure and morphology, X-ray analysis, Raman spectrometry and transmission electron microscopy (TEM) were also performed. In vitro NLC cytocompatibility was assessed on two fibroblast cell lines, one murine (NIH-3T3) and one human (HFF1). Finally, mucoadhesive properties were evaluated on the selected NLC, optimized by the addition of a coating layer of the positively charged cationic lipid didodecyldimethylammonium bromide (DDAB).

## 2. Materials and Methods

### 2.1. Materials

Kolliphor RH40 was provided by BASF Italia S.p.a. (Cesano Modena, Italy). Oleoyl Macrogol-6 Glycerides (Labrafil) was gifted by Gattefossé Italia s.r.l. (Milano, Italy). Hydrogenated Coco-Glycerides (Softisan 100) was bought from IOI Oleo GmbH (Oleochemicals, IOI group). *Rosmarinus officinalis* L. and *Lavandula × intermedia* “Sumian” essential oils were provided by Exentiae s.r.l. (Catania, Italy). *Mentha piperita* was gifted by the Department of Agriculture, Food and Environment (Di3A), University of Catania. Triglyceride caprylic-capric (Tegosoft CT, Miglyol 812) was supplied by Farmalabor (Canosa di Puglia, Italy). Neem oil was supplied by La Saponaria (Vallefoglia, Italy). Dioctadecylammonium bromide (DDAB), mucin from porcine stomach type II, sodium chloride, potassium chloride, calcium chloride dehydrated, and polysorbate 80 were bought from Sigma (Milan, Italy).

### 2.2. Nanoparticles Preparation

NLC were produced by the phase inversion temperature (PIT) method, as previously described [40]. The lipid phase containing the surfactant mixture (6.0% *w/v* of Kolliphor RH40 and 7.5% *w/v* of Labrafil) and the lipid Softisan (10% *w/v*) and the aqueous phase were separately heated to 70 °C. Then, the EO was added to the lipid phase at 1:1 ratio with the solid lipid. Finally, the aqueous phase was added dropwise to the lipid phase and the mixture was rapidly cooled in an ice bath under stirring for 1 h, thus obtaining different NLC formulations: L-NLC, containing *Lavandula*; M-NLC, prepared with *Mentha*; R-NLC, using *Rosmarinus*. Tegosoft CT-loaded NLC (CT-NLC) were prepared as positive control, while Neem oil was used to obtain N-NLC, as negative control. The produced NLC were purified through ultracentrifuge (SL16R Centrifuge, Thermo Scientific, Rodano, Italy) at 13,000 rpm for 2 h at 1 °C, in order to remove the excess surfactants from the colloidal suspensions. The obtained pellet was vortexed (Heidolph Reax 2000, VWR, Milan, Italy) for 60 s.

### 2.3. Photon Correlation Spectroscopy (PCS)

After 24 h from the preparation, samples were diluted 1:10 in deionized water and analyzed using a Zetasizer Nano S90 (Malvern Instruments, Malvern, UK), in order to measure the mean particle size (Zave), the polydispersity index (PDI), and the zeta potential (ZP). The obtained values were reported as the mean of at least three measurements ± standard deviation (SD).

### 2.4. Osmolarity and pH Measurement

The osmolarity of each formulation was detected using an osmometer (Osmomat 3000, Gonotec, Berlin, Germany) previously calibrated with ultra-purified water and physiological solution. Moreover, the pH value of each formulation was measured through a pH-meter (Mettler Toledo, Milano, Italy).

### 2.5. Entrapment Efficiency

In order to quantify the entrapment efficiency (EE%) of each EO, all formulations were centrifuged at 13,000 rpm, 4 °C for 2 h. The separated supernatants were diluted with an ethanol–water mixture (75:25) and analyzed using a UV–vis spectrophotometer (UH5300 UV-Visible Double-Beam Spectrophotometer, Hitachi Europe, Milan, Italy). The used wavelengths were 228 nm for *Rosmarinus* EO, 230 nm for *Mentha* and *Lavandula* EO, and 224 nm for Neem oil. The calibration curves used for the quantitative evaluation of each oil were linear, considering the following ranges: 2–0.06 mg/mL (R^2^ = 0.9927) for *Rosmarinus* EO; 2–0.03 mg/mL (R^2^ = 0.9960) for *Mentha* EO; 2–0.03 mg/mL (R^2^ = 0.9865) for *Lavandula* EO; 2–0.06 mg/mL (R^2^ = 0.9915) for Neem oil. Entrapment efficiency was determined using the following equation:

(1) $$\mathrm{EE}\%=\frac{\left(\mathrm{total}\;\mathrm{amount}\;\mathrm{of}\;\mathrm{oil}\;\mathrm{used}-\mathrm{amount}\;\mathrm{of}\;\mathrm{unencapsulated}\;\mathrm{oil}\right)}{\mathrm{total}\;\mathrm{amount}\;\mathrm{of}\;\mathrm{oil}\;\mathrm{used}}\times 100$$

### 2.6. Stability Studies by Turbiscan^®^ AG STATION

The optical analyzer Turbiscan^®^ Ageing Station (TAGS, Formulaction, L’Union, France) was used to assess the formulations physical stability, as it has been demonstrated to be reliable in the analysis of aggregation and/or migration instability phenomena [40,41,42,43,44,45]. In our experiment, 20 mL of each sample was inserted into a cylindrical glass cell, stored at 25.0 ± 1.0 °C and analyzed for 30 days. For detailed explanation of the analyzer functioning, we remand to the literature [15]. The variation of backscattering profiles (ΔBS) was used to compare the formulations, considering the Turbiscan^®^ Stability Index (TSI), which numerically quantifies the formulation’s stability.

### 2.7. X-ray Analysis

The assessment of X-ray diffraction of the samples was carried out with a MiniFlex Rigaku diffractometer, operating in step-scan mode and equipped with a Cu Kα source (wavelength λ = 0.154 nm) at 30 kV and 100 mA. The X-ray diffraction data were collected in the Bragg–Brentano geometry, from 5 to 35 deg, at a scanning speed of 0.02 deg/s. Crystallinity index values, related to the intensity of the peak obtained, were calculated following the equation below:

(2) $$C=\frac{S_{\mathrm{tot}}-S_{a}}{S_{\mathrm{tot}}}$$

where S_tot_ is the total area under the graph and S_a_ is the area subtended to the only amorphous region.

### 2.8. Raman Spectrometry

As previously described [15,46], Raman spectroscopy analysis was carried out using a micro-Raman spectrometer (INVIA, Renishaw, Gloucestershire, UK), which includes a 514.5 nm air-cooled Argon ions laser source and an 1800 lines/mm grating monochromator/grid polychromator with RenCam CCD detection, with a resolution of 1 cm^−1^. The laser source was focused on the suspension of NLC with 100× long working objectives (a long working distance) to a stop diameter of about 1 μm. The acquisition time of Raman spectra varied according to the intensity of the Raman signals and lasted until a satisfactory signal-to-noise ratio was reached. Data analysis was performed by using Renishaw Wire 2.0 software. The results are reported as the mean of the intensity of 100 accumulation spectra acquired from 5 different regions, with a spatial resolution of 5 microns in each sample.

### 2.9. Morphological Analysis by TEM

In order to analyze the morphology of the produced nanoformulations, transmission electron microscopy (TEM) analysis was performed. The samples were prepared placing 5 μL of each NLC on a 600-mesh carbon coated copper grid (TAAB Laboratories Equipment, Berks, UK), adding a drop of 2% *w/v* aqueous solution of gadolinium triacetate (Uranyl Acetate Alternative), for 2 min, and finally drying at room temperature after the removal of the exceeding solution. The analysis was carried out through a transmission electron microscope (model HITACHI) operating at 100 KV acceleration voltage.

### 2.10. In Vitro Release Study

*Lavandula* and *Rosmarinus* release from L-NLC and R-NLC, respectively, was evaluated by using Franz-type diffusion cells (LGA, Berkeley, CA, USA). Before being mounted in Franz-type diffusion cells, 0.75 cm^2^ regenerated cellulose membranes (Spectra/Por CE; Mol. Weight Cut-off 3.5 kDa) were moistened by immersion in water for 1 h at room temperature. The receptor compartment was filled with 4.5 mL of simulated nasal electrolytic solution (SNES) (sodium chloride 0.745 g, potassium chloride 0.129 g, calcium chloride dehydrated 0.005 g, and distilled water q. s. 100 mL) containing 0.5% of polysorbate 80, to reach the sink conditions [47], thermostated at 37 °C, and constantly stirred at 600 rpm. Then, 500 µL of each sample was applied in the donor compartment. The experiments were run for 48 h. At scheduled time intervals (0, 1, 2, 3, 4, 5, 6, 7, 8, 9, 24, and 48 h), 200 µL of the receptor medium was withdrawn and replaced with an equal volume of medium equilibrated to 37 °C. Each sample was analyzed by the UV method described in Section 2.4 to determine the EO content.

### 2.11. Cytocompatibility Assay

The cytocompatibility was assessed on two fibroblast cell lines, the NIH 3T3 mouse embryonic and the HFF1 human cell lines. NIH 3T3 and HFF1 were cultured in Dulbecco’s modified Eagle’s Medium (DMEM, ATCC, Manassas, VA, USA) supplemented with 10% v/v fetal bovine serum and (50 IU/mL) penicillin/(50 µg/mL) streptomycin, in a controlled environment with a temperature of 37 °C, 5% CO_2_ concentration, and 95% relative humidity [48]. The cells were seeded in a 96-well plate (1 × 10^4^ cells/well) and incubated for 24 h to allow adherence. EO-NLC were diluted in DMEM in order to test different concentrations (100, 200, 400, and 500 μg/mL) and the cells were treated for 24 h. The two cell lines were also treated with DMEM medium alone, as negative control, and vehicle (Tris buffer) as positive control. The cell viability was evaluated by adding 200 µL of MTT (0.5 mg/mL) in culture medium to each well and incubated for 2 h. The optical density (OD) was measured with a microplate spectrophotometer reader (Synergy HT multi-mode microplate reader, BioTek, Milano, Italy) at λ 550 nm. The results are expressed as percentage of cell viability with respect to untreated control viable cells, whose value was equal to 100%.

### 2.12. Mucoadhesive Evaluation of NLC

Positively charged NLC (L-NLC+, R-NLC+, and CT-NLC+), respectively, prepared with *Lavandula*, *Rosmarinus,* or Tegosoft CT, were obtained as previously described [49], adding 0.15% w/w of the cationic lipid DDAB to the oily phase. An in vitro method based on the evaluation of two parameters (zeta potential and turbidimetry) was used to assess the mucoadhesive properties of NLC. Briefly, mucin (0.1% *w/v*) was suspended in PBS (pH 5.8) and stirred overnight to allow its complete dispersion. The interaction between each positively charged NLC and mucin was determined by mixing equal volumes of mucin dispersion and NLC for 15 min at 25 °C. After 1 and 24 h of incubation at 37 °C, zeta potential and turbidimetric measurements were performed. In particular, turbidimetric measurements were evaluated comparing the absorbances at 650 nm by UV–Vis spectrophotometer of the native mucin and each dispersion.

### 2.13. Statistical Analysis

All data are reported as mean values ± SD. Differences, analyzed by two-sample hypothesis testing (*t*-test), using Origin Software (version 8.5.1), were considered statistically significant for *p* < 0.05. For cytocompatibility studies, statistical differences among treatments were assessed by one-way ANOVA followed by Bonferroni test.

## 3. Results and Discussions

### 3.1. Physicochemical and Technological Characterization

**Figure 1:**
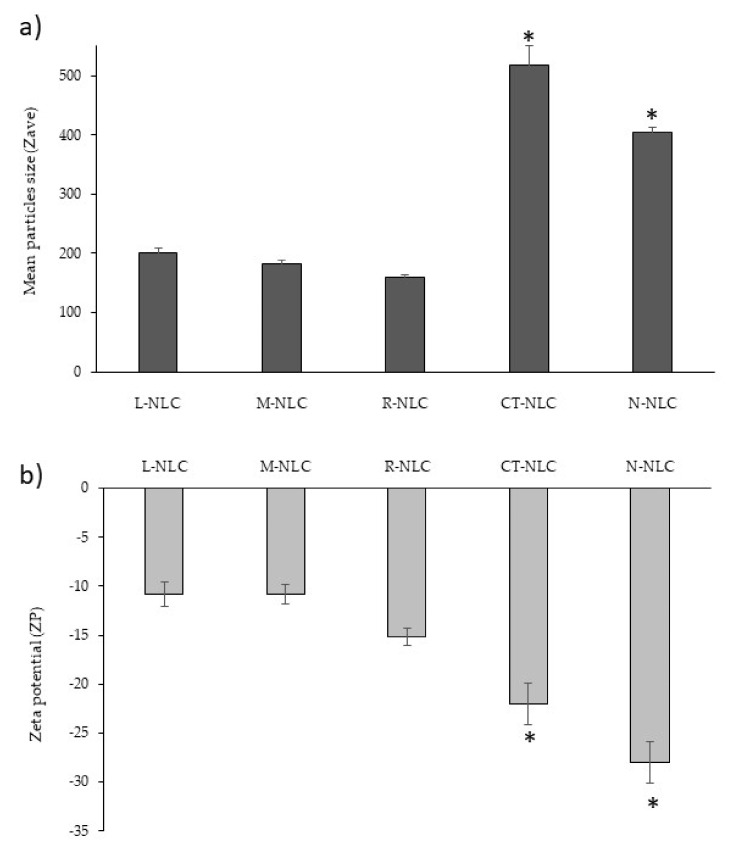
(**A**) Mean particle size (Zave, nm) and (**B**) Zeta Potential (ZP) ± standard deviation (SD) of the prepared Lavandula NLC (L-NLC), Mentha NLC (M-NLC), Rosmarinus NLC (R-NLC), Tegosoft CT NLC (CT-NLC) and Neem NLC (N-NLC). * Significance for *p* < 0.05, comparison between EO-loaded NLC (L-NLC, M-NLC, and R-NLC) and the control NLC (CT-NLC and N-NLC).

EO-loaded NLC for intranasal administration were produced by the PIT method, previously reported as a green technology that allows reducing the amount of surfactants and energy required [40]. All NLC were analyzed 24 h after their production in order to evaluate their mean size, polidispersity, zeta potential, osmolarity, pH, and the EE% of the EO. PCS results (Figure 1) show that NLC prepared with *Lavandula*, *Mentha,* or *Rosmarinus* showed the presence of small-sized nanoparticles of about 200 nm (Figure 1), therefore adequate for the intranasal route [25]. In particular, EO-loaded NLC were smaller compared to those prepared using Tegosoft CT or Neem oil, which also showed higher PDI values related to the presence of heterogeneous nanosuspensions. It is worth noting that *Lavandula*, *Mentha* or *Rosmarinus* favor the formation of nanoparticles with great homogeneity, as confirmed by the low PDI, a relevant parameter that strongly affects NLC feature and stability [50]. Indeed, PDI values were found to be lower than 0.3, therefore related to the presence of a single peak of size distribution, in agreement with previous data [4].

In order to ensure the formulation’s safety on cell functioning, viability, and stability, without potential damages and alterations due to water movements through the membranes, it is important that NLC osmolarity and pH values are kept in the physiological range [51]. As reported in Table 1, osmolarity values of the produced NLC were in the range 291–299 mOsm/kg. In addition, pH measurements showed that pH values were in the physiological range for all nanosuspensions, with values between 7.09 and 7.28 for all formulations. It is interesting to highlight that *Lavandula*, *Mentha,* and *Rosmarinus* showed significantly higher EE% values compared to Neem oil (Table 1). As expected, a certain loss of EO was observed, due to the presence of micelles of surfactants, which do not participate in the formation of nanoparticles. For this reason, each system was further analyzed after the purification step.

These findings, in agreement with PCS results, would suggest a better organization of the raw materials at the interface when using the three EOs, as also suggested by the smaller particles obtained for L-NLC, M-NLC, and R-NLC.

As shown in Figure 1, all samples were negatively charged (≈−20 mV), without significant differences related to the oil, even if the presence of Neem oil induced a slight increase in ZP value, confirming literature findings [52]. As reported in the literature [53], high ZP values would suggest a long-term physical stability of the colloidal suspension, due to nanoparticles’ repulsions.

**Figure 2:**
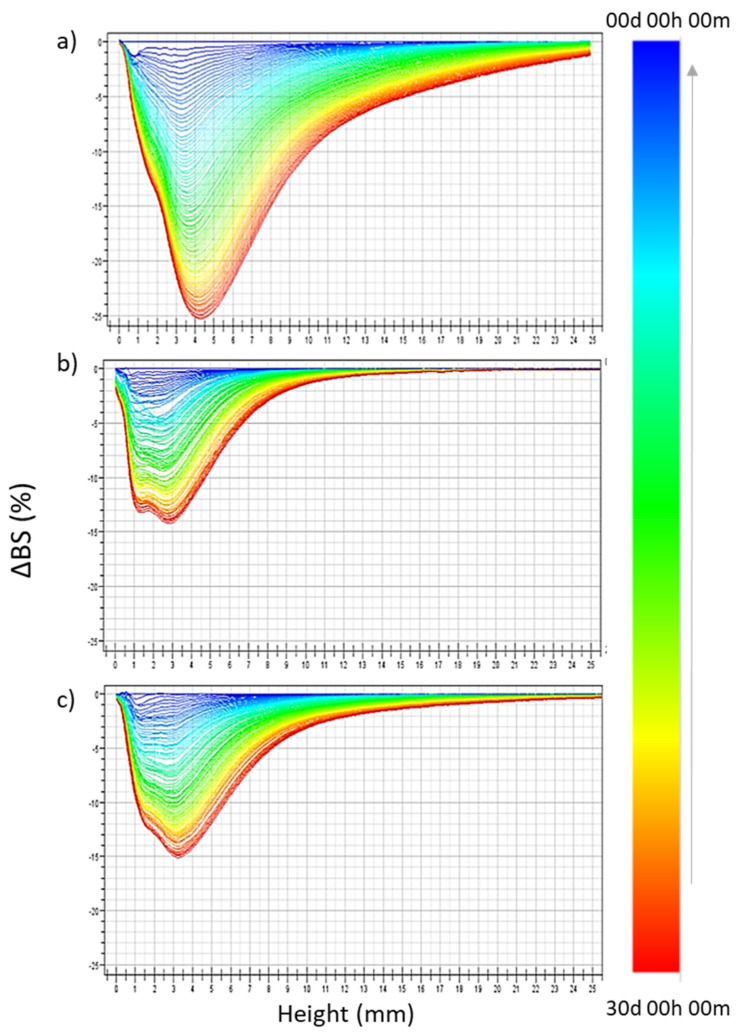
Backscattering profiles (ΔBS) of CT-NLC (**a**), L-NLC (**b**), and N-NLC (**c**) stored in Turbiscan^®^ for 30 days at a temperature of 25.0 ± 1.0 °C. Data are presented as a function of time (0–30 days) of sample height (0 to 20 mm) (the direction of analysis time is indicated by the arrow).

In order to deepen the NLC stability, a key parameter for nano-sized colloids [50], we exploited Turbiscan^®^ technology, storing samples for 30 days at 25 °C. Interestingly, the instability kinetics obtained by TSI values, as reported in Table 2, showed that NLC prepared with the three EOs were more stable compared to CT-NLC, which showed a significant increase already after 3 weeks of storage. In particular, the following decreasing scale of stability can be described as follows: L-NLC = R-NLC ≥ N-NLC ≥ M-NLC >> CT-NLC. Therefore, NLC prepared with *Lavandula* and *Rosmarinus* showed a greater stability compared to other samples, probably due to the smaller mean size and the higher homogeneity of the nanoparticles in suspensions. Among all the prepared formulations, the use of the commercial oil CT reduced the colloidal suspension’s stability, as confirmed by the backscattering variation reported in Figure 2a. Herein, significant instability phenomena related to both particle migration and aggregation occurred (ΔBS ≥ 20%).

The greater stability of L-NLC and N-NLC was confirmed by the absence of significant variation in BS profiles, as resulted by the low values registered, related to slight particle migration at the bottom of the cuvette (Figure 2b,c). R-NLC and M-NLC showed similar profiles to those reported for L-NLC and N-NLC, respectively (data not reported).

The stability results are in agreement with PCS measurements of samples analyzed after 28 days of storage, showing that, at the 0.05 level of significance, the difference between the population mean size and PDI was significantly different for mean size only for CT-NLC, whose particles were found to be of about 500 nm (Figure S1). These data are in agreement with previous findings, with L-NLC showing a more stable long-term behavior thanks to the tendency of the nanoparticles to agglomerate, thus determining the formation of a flocculated suspension, whose nanoparticles can be easily dispersed by gentle shaking.

**Figure 3:**
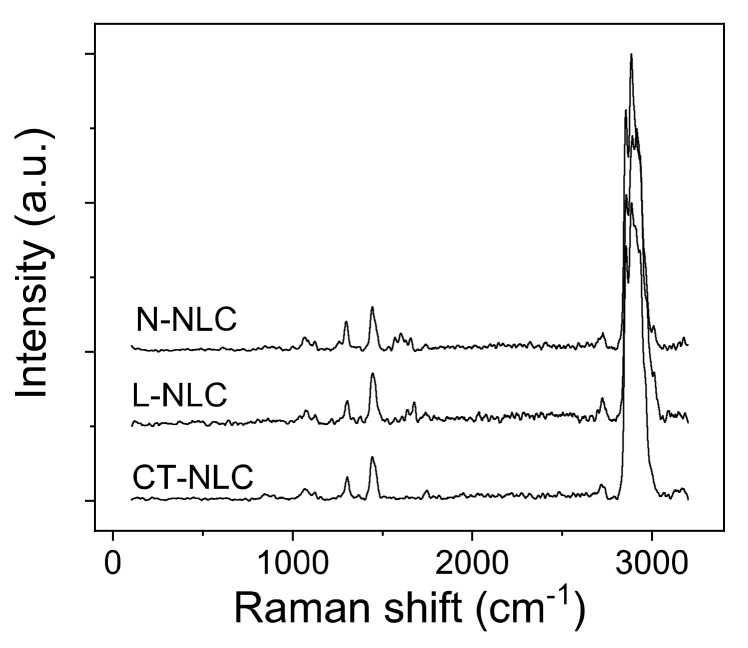
Raman spectra of L-NLC, CT-NLC, and N-NLC.

In order to analyze the influence of the oily component on the NLC structure, X-ray diffraction, Raman spectroscopy, and Transmission Electron Microscopy (TEM) were performed. In particular, NLC were analyzed through Raman spectroscopy using a micro-Raman spectrometer, in order to obtain the so-called “molecular footprint” of the sample, which provides information about the sample’s molecular composition and structure [54]. In particular, through molecular vibrations produced by a laser beam, a map of chemical and structural changes in molecules can be obtained, which describes conformation and arrangement in lipid chain after the addition of the oil [55]. Since a similar behavior was observed for NLC produced using Neem oil or *Mentha* EO compared to NLC prepared using *Lavandula* or *Rosmarinus* EO, for a clearer representation of data, we report only L-NLC and N-NLC Raman spectra vs. CT-NLC (Figure 3). The Raman spectra of all the prepared NLC are reported in Figure S2. Although no shifts in the frequencies of the Raman transitions arise in a comparison of the different samples, the relative intensities in significant regions change conspicuously for L-NLC compared to controls, CT-NLC and N-NLC (Figure 3).

The most intense bands in the spectra of molecules including alkanes and lipids with alkyl groups are the CH stretching modes. The most common vibrational Raman active modes in the analyzed systems are summarized in Table 3. Both frequency differences and relative intensity changes for these vibrational modes have been used to monitor specific conformational changes in the hydrocarbon chains [56]. The 1100 cm^−1^ region in particular has been shown to be a superposition of the C-C stretching modes for segments of all-trans hydrocarbon conformations.

An increase in intensity of the 1115 cm^−1^ band relative to the intensities of the 1050 cm^−1^ transitions (I_1115_/I_1050_) is indicative of a greater fluidity within the hydrocarbon chains; therefore, the increase in the 1115 cm^−1^ band originates from the increased intramolecular disorder in the systems. The Raman spectra of the NLC systems in the 2750–3050 cm^−1^ spectral region, i.e., the C-H stretching vibration region, are well fitted as a sum of six Gaussian bands, as reported in the Table S1. It is well known that lipids interact with phospholipidic membrane. De Lange et al. [57] reported that Raman spectra of binary vesicles–cholesterol bilayers showed little variation associated with cholesterol, which allows spectral changes to be assigned to the lipid-chain vibrational signatures of the liquid-disordered (l-d), solid-ordered (s-o), and liquid-ordered (l-o) phases. Analyzing the C–H stretching, C=O stretching, and CH_2_ bending modes, it is possible to monitor the structural order evolution of cholesterol in vesicle systems [58]. The region around 3000 cm^−1^ of the Raman spectrum consists of a large number of overlapping peaks, containing both fundamental CH-stretch vibrations and Fermi resonance bands. The CH_3_ symmetric stretching modes appear in the 2870–2880 cm^−1^ spectral region, with a Fermi resonance (FR) component in the 2930–2940 cm^−1^ region. The peaks in the 2950–2970 cm^−1^ spectral region are the CH_3_ out-of-plane and in-plane methyl antisymmetric stretches. The methylene vibrations at approximately 2850, 2880, 2900, and 2930 cm^−1^ are sensitive to conformational changes as well as intermolecular interactions of the alkyl chains of lipids. The νa(CH_2_) antisymmetric stretch is coupled to rigid rotations–torsional vibrations, so that it broadens considerably with temperature, and increases continuously in frequency, from 2880 to 2900 cm^−1^, as gauche conformers are introduced. The νs(CH_2_) symmetric stretch contains three components, centered at 2852, 2900, and 2928 cm^−1^, because of extensive Fermi resonance interactions with overtones of the bending modes, and it is affected by intra- and inter-molecular interactions. The relative intensities of the peaks in this last spectral region change notably with changes in hydration state, packing, and conformational order [57,58]. In order to exploit this spectral sensitivity toward the lipid environment, several spectral parameters have been used in the literature that empirically describe the order of the lipid matrix. The peak height ratio I_2890_/I_2850_ has been used as a marker for chain packing and conformational disorder, where higher values indicate a higher ordering of the chains [59]. Table 4 resumes the Raman intensity ratios related to C-C stretching vibrational bands (I_1115_/I_1050_) and C-H stretching vibrational bands I_2890_/I_2850_ for all samples.

**Figure 4:**
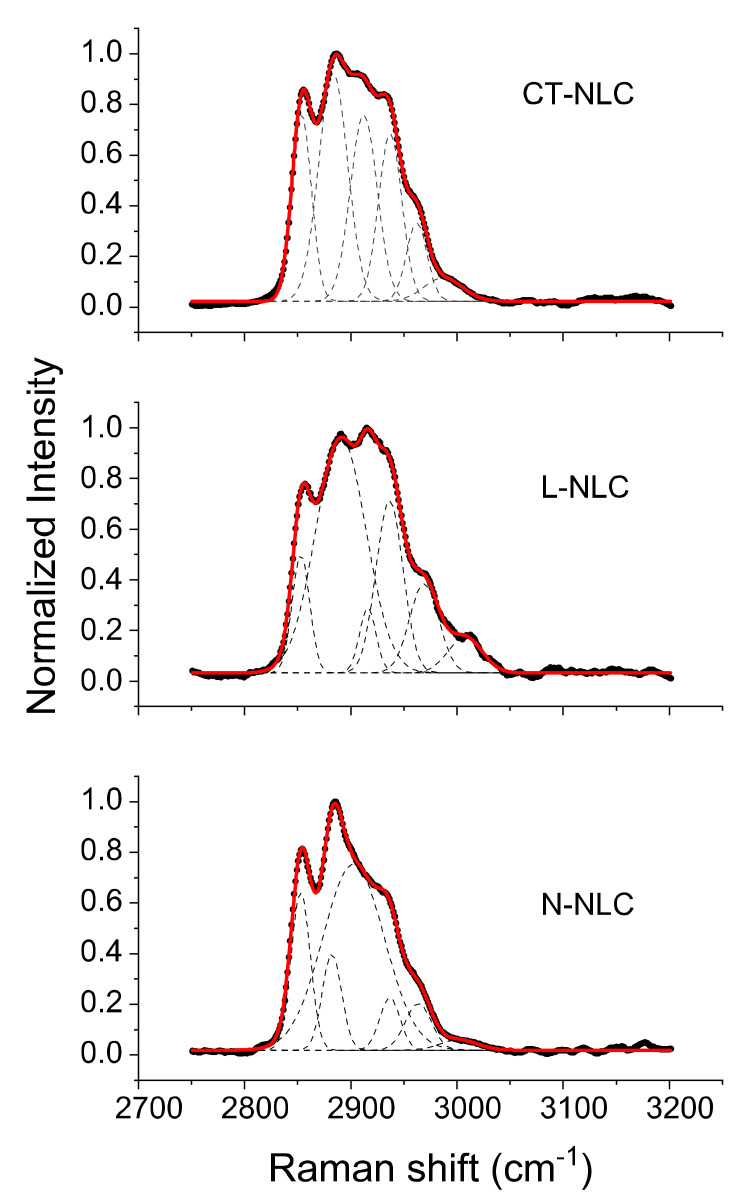
Raman spectra of NLC prepared with Lavandula (L-NLC), Tegosoft CT (CT-NLC), and Neem oil (N-NLC): region around 3000 cm^−1^.

The Raman spectra of L-NLC, CT-NLC, and N-NLC systems in the C-H stretching vibration region are shown in Figure 4. The spectrum in the 2750–3050 cm^−1^ spectral region is well fitted as a sum of six Gaussian bands, as reported in the Supplementary Materials.

**Figure 5:**
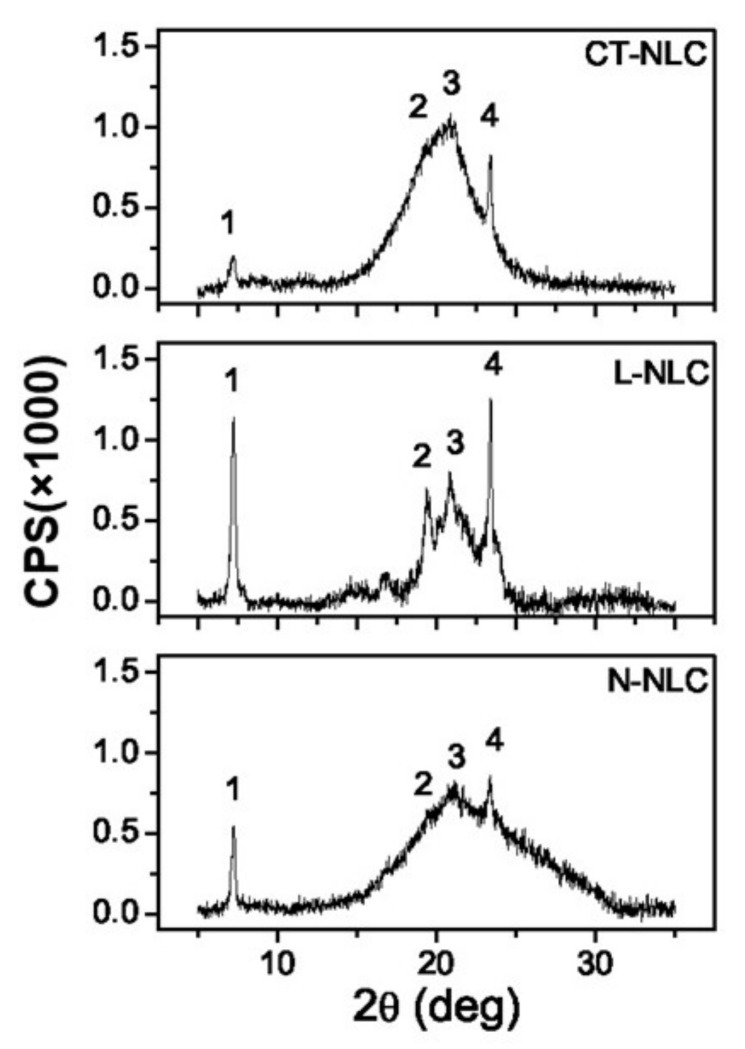
XRD spectra of representative samples L-NLC, CT-NLC, and N-NLC.

XRD measurements were performed for all NLC, showing a similar behavior between *Lavandula*, *Mentha* and *Rosmarinus* with respect to the controls CT-NLC and N-NLC. Figure 5 shows the XRD spectra recorded for L-NLC vs. control NLC samples. The presence of discrete peaks, more or less pronounced, emerging from a very large peak is evident in each spectrum. This indicates that the observed material is characterized by a semi-crystalline structure. The discrete peaks (labeled 1–4) correspond to the interplanar spacings of 1.22, 0.46, 0.42, and 0.38 nm, respectively. These four peaks are clearly visible for L-NLC. M-NLC and R-NLC showed similar XRD spectra to that of L-NLC (Figure S3). Contrarily, CT-NLC and N-NLC showed a considerably reduced intensities of Peaks 2 and 3. The occurrence of such peaks indicates the presence of ordered solid lipids in the β and β’ crystallographic modifications [60]. In addition, in Figure 4, it is evident that the relative intensity of the various peaks is different for each sample: the peaks are well pronounced in all EO-loaded NLC, as reported for L-NLC (Figure 5), but are very low in the controls CT-NLC and N-NLC.

Through X-ray diffraction, it was possible to measure the NLC crystallinity index (C), an important parameter that allows predicting polymorphic transitional changes during storage [55]. Crystallinity was measured between 5 and 30 degrees, and the obtained results are reported in Table 5. Comparing the NLC produced with different EOs, it emerged that the use of *Lavandula* and *Rosmarinus* led to the formation of NLC with the highest values of crystallinity (68% and 40%, respectively), while NLC prepared using *Mentha* or Neem oil showed lower values (30% and 20%, respectively). It is possible that the high amount of linalool present in the complex mixture of *Lavandula* and *Rosmarinus* increases the NLC stability, as we previously reported [4]. Interestingly, the lowest crystallinity value (10%) was registered for CT-NLC, related to the presence of a less ordered structure. As reported in the literature, the crystallinity of the lipid matrix is affected by the structure of the liquid oil [61]. Therefore, the following scale based on the reduction of the crystallinity value can be described: L-NLC > R-NLC > M-NLC > N-NLC > CT-NLC. The lowest crystallinity found for caprylic/capric triglyceride is consistent with literature findings, in which the addition of a synthetic oil was demonstrated to strongly reduce the crystallinity of the lipid matrix [61,62]. It is interesting to highlight that the crystallinity scale presents the same order of the stability scale obtained by Turbiscan^®^ analysis, with L-NLC and R-NLC as the most stable formulations compared to other NLC.

**Figure 6:**
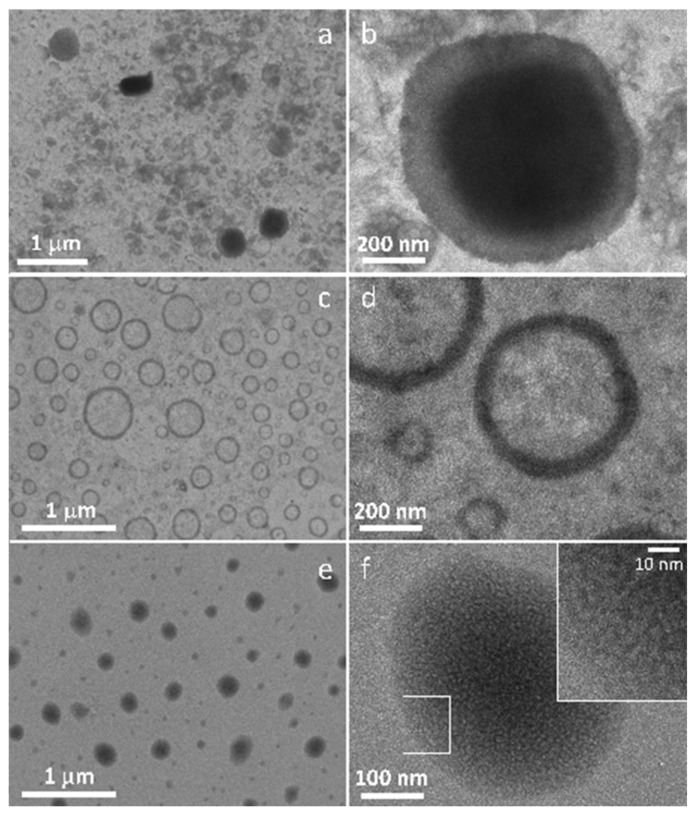
Transmission electron microscopy (TEM) images of: CT-NLC (**a**,**b**); N-NLC (**c**,**d**); L-NLC (**e**,**f**).

In order to analyze the NLC morphology, TEM analysis was performed. Figure 6 reports TEM images of L-NLC, CT-NLC and N-NLC. In particular, CT-NLC (Figure 6a,b) showed a very irregular arrangement of nanoparticles, with a central oily core and a large shell. N-NLC (Figure 6c,d) and M-NLC (Figure S4) showed the presence of a faint central oily core surrounded by the solid lipid and surfactants layers, in agreement with previous studies [63,64]. On the other side, L-NLC (Figure 6f) and R-NLC (Figure S5) showed a structure characterized by the presence of many oily droplets dispersed in the solid lipid matrix, in agreement with previous findings [65]. It is possible that the ability of *Lavandula* and *Rosmarinus* to create imperfections in the NLC matrix also affects the nanoparticles’ structure, improving their order and, consequently, stability during storage, probably due to their composition characterized by high amount of linalool [4,15,16].

**Figure 7:**
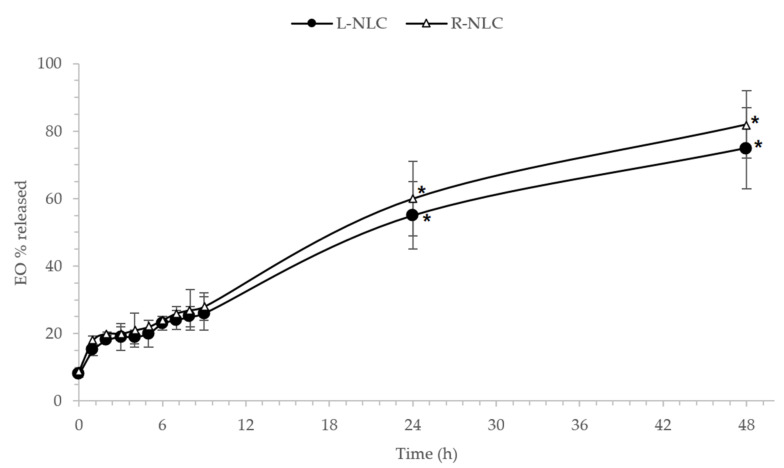
Percentage of Lavandula and Rosmarinus EOs released from L-NLC and R-NLC, respectively, at different time intervals up to 48 h. Each value is the mean of six independent experiments. * Significance for *p* < 0.05.

As reported in Figure 7, the release profiles of EOs from NLC was similar for Lavandula and Rosmarinus, with a sustained release over the first 8 h, with about 20% of EO released from NLC. After 24 and 48 h, the cumulative amount of EO reached about 50% and 80% of the loaded oil, respectively (Figure 7). Our results are in agreement with previous findings [66], confirming the advantages of EO encapsulation into NLC that would provide a sustained and prolonged release.

### 3.2. In Vitro Cytocompatibility

**Figure 8:**
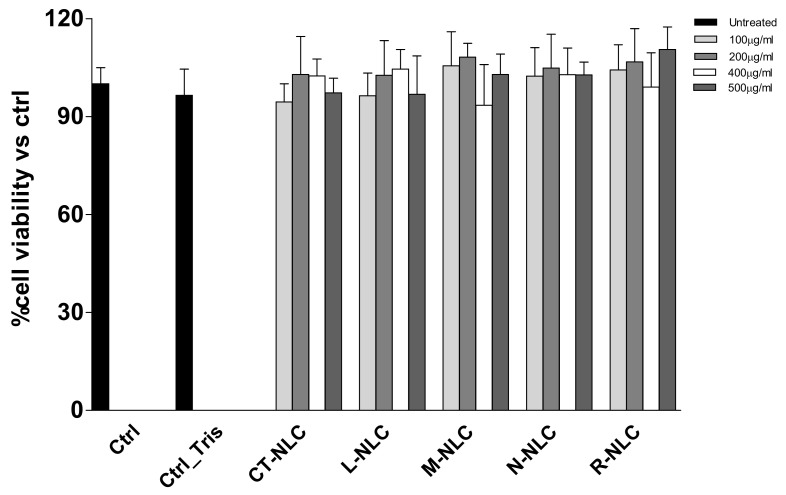
Murine fibroblasts NIH-3T3 viability after 24 h treatment with different concentrations of Tegosoft CT NLC (CT-NLC), Lavandula NLC (L-NLC), Rosmarinus NLC (R-NLC), Mentha NLC (M-NLC), and Neem NLC (N-NLC). All values are mean ± SD of three experiments in triplicate.

**Figure 9:**
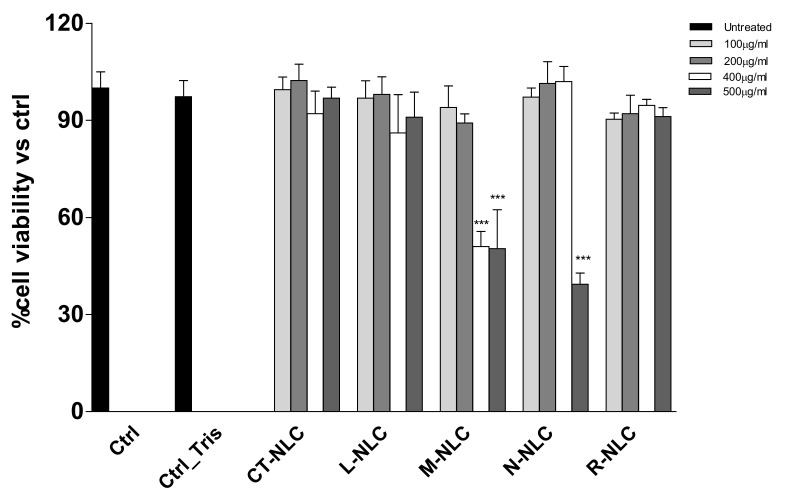
Human fibroblasts HFF1 viability after 24 h treatment with different concentrations of Tegosoft CT NLC (CT-NLC), Lavandula NLC (L-NLC), Rosmarinus NLC (R-NLC), Mentha NLC (M-NLC), and Neem NLC (N-NLC). All values are mean ± SD of three experiments in triplicate. *** *p* < 0.001 vs. control.

3-(4,5-dimethylthiazol-2-yl)-2,5-diphenyltetrazolium bromide (MTT) assay was performed in order to assess NLC cytocompatibility. Some NAD(P)H-dependent oxidoreductase enzymes are produced by living cells and are able to cause the conversion of yellow MTT salt into purple formazan. The intensity of the color is directly related to the cells’ viability; quantification of formazan crystals is carried out through absorbance measurement. MTT assay was performed on human (HFF1) and murine (NIH-3T3) fibroblast cell lines, selected to test the biocompatibility and safety of NLC nanosuspensions, which is essential to ensure that the NLC do not affect the capability of normal cells to produce trophic growth factor, which support a variety of cellular processes of the above epithelial tissues [67]. All the prepared NLC were tested at different concentrations (100, 200, 400, and 500 μg/mL) and compared to controls. The results are reported in Figure 8 and Figure 9.

As expected, the control formulation prepared using Tegosoft CT (CT-NLC), did not affect cell viability of both murine and human fibroblasts, showing a cell viability higher than 90% at all tested concentrations. No cytotoxic effects were observed when murine fibroblasts were treated with N-NLC, while the highest concentration (500 μg/mL) significantly reduced human fibroblast viability by a hefty 60%. This result is supported by previous studies showing Neem cytotoxic effects on different cell lines (embryonic 3T3 fibroblasts, HeLa tumor cells, HaCat keratinocytes, and V79-4 pulmonary fibroblasts) when used at low concentration and in combination with oleic acid [63]. Furthermore, Neem oil was found to induce cell death in a time-dependent manner on HCT116 cells, whose effect was attributed to the cell cycle arrest and apoptosis due to Neem limonoids, even if the molecular mechanism of Neem limonoid-induced cell death has not been described [68]. Interestingly, L-NLC and R-NLC showed good biocompatibility on both murine and human cell lines, at all tested concentrations. This result is consistent with previous studies demonstrating that the EO encapsulation is able to improve the oil biocompatibility on Raw 264.7 cells (macrophage cell line) [4]. *Mentha* EO-loaded NLC (M-NLC) showed a dose-dependent effect, since concentrations equal to 100 and 200 μg/mL resulted to be highly biocompatible, while a 50% reduction in fibroblasts viability was observed at 400 and 500 μg/mL. This result is consistent with earlier research, in which *Mentha* species EOs have been reported to exert cytotoxic effects that can be exploited to treat cancer, due to their ability to inhibit the cell proliferation of numerous tumor cells by acting on mitochondrial dysfunctions, apoptosis induction, and autophagy processes [69,70].

**Figure 10:**
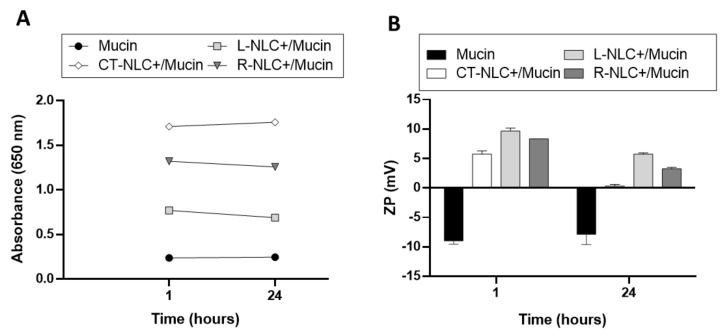
In vitro assessment of mucin interactions with CT-NLC+, R-NLC+, and L-NLC+ by turbidimetric assay at 650 nm (**A**) and Zeta potential (ZP) modifications (**B**).

Based on the obtained results, *Lavandula* and *Rosmarinus* were selected as safe and promising EOs to be encapsulated into NLC for the potential treatment of Alzheimer’s disease, thanks to the formation of small and homogeneous particles and a more ordered structure related to the formation of oily droplets into the lipid matrix. In order to exploit the intranasal delivery, we aimed at improving the mucoadhesive properties of the systems, with the addition of a coating layer of the cationic lipid DDAB, thus obtaining L-NLC+, R-NLC+, and CT-NLC+, respectively, prepared with *Lavandula*, *Rosmarinus,* and Tegosoft CT. DDAB at 150 µg/mL was selected as a safe concentration able to guarantee positive ZP value. As recently reported, DDAB is safe on human keratinocytes (HaCaT) and osteoblast-like (SAOS-2) cell lines, with cell viability being higher than 90% of control at 165 µg/mL and higher than 50% for twice this concentration (330 µg/mL) [71]. As expected, the addition of such a low amount of the positively charged coating layer did not induce significant modification in mean size, whose values were found to be 211.1 and 184.5 nm for L-NLC and R-NLC, respectively, without affecting PDI (<0.3). ZP was found to be highly positive, with values equal to +40.5 and +43.0 mV for L-NLC and R-NLC, respectively. In order to verify the mucoadhesive properties of L-NLC+ and R-NLC+, compared to the control CT-NLC+, the interaction with mucin was evaluated over a period of 24 h, by measuring the turbidity at 650 nm (ABS) and the change in the ZP values (Figure 10).

Absorbance measurement was reported to give a rough estimation of particle–mucin interaction [72]. The mucoadhesive interaction between particles and mucin results in the adsorption of the mucin around the surface of the particles with a consequent slight aggregation that can be detected as an increase in UV absorbance [73]. For this reason, the turbidity value after mixing CT-NLC+, R-NLC+, and L-NLC+ with native mucin was determined after 1 h and followed up to 24 h. As shown in Figure 10A, the turbidity of all NLC+/mucin dispersions was higher than the turbidity of mucin dispersion itself at both the time points considered, thus suggesting the interaction between NLC+ and mucin [74]. Furthermore, high absorbance values of CT-NLC+, R-NLC+, and L-NLC+ dispersions before incubation with mucin (whose values were 2.015, 1.226, and 2.049, respectively) reflected particle motion [72], while reduced values after their incubation with mucin (Figure 10A) are related to possible particle immobilization due to adsorption of mucin on their surface [75]. As reported by D’Angelo et al., mucin’s adsorption on particle surface is expected to reduce particle mobility, agglomeration, and to a certain extent precipitation might take place [76]. Our results suggest higher interactions for both nanosystems with EO compared to the CT-NLC+.

Particle–mucin interactions were further confirmed by ZP measurements. Important changes in ZP value are related to strong mucoadhesive properties. In our previous study, the interaction between mucin and PLGA-PEG nanoparticles (NPs) intended for intranasal administration was evaluated [77]. Herein, ZP of PLGA-PEG NPs remained almost unchanged in the presence of mucin, demonstrating that weak interactions occurred between PEGylated NPs and mucin [77].

In the present study, upon addition of CT-NLC+, R-NLC+, and L-NLC+ to mucin, the negative ZP of mucin (~−9 mV) was inverted to a positive value (Figure 10B), and consequently a significative variation in the ZP of CT-NLC+, R-NLC+, and L-NLC+ (~+20 mV) was observed after incubation with mucin owing to the mucin coating. According to turbidimetric results, the interaction was higher for NLC+ with EOs in the following order: L-NLC+ > R-NLC+ > CT-NLC+.

Mucins are highly glycosylated glycoproteins with a large peptide backbone and oligosaccharides as side chains. Their protein backbone is characterized by the presence of repeating sequences rich in serine, threonine, and proline residues. The net negative charge is due to the presence of deprotonated carboxylate groups (sialic acid) and ester sulfates at the terminus of some sugar units [78]. The mucoadhesive study was carried out at a specific pH value (pH 5.8) according to the intranasal administration route. The loss of electrostatic interaction of mucin at low pH led to a conformational change from a random coil to a rod-like structure by exposing hydrophobic regions, which were folded and sequestered in the inner part at neutral pH. This is a favorable condition for the interactions between mucin and other entities [77,79]. The electrostatic interaction is the most expectable mucoadhesive mechanism. This may be due to the interactions between the negatively charged sialic groups of mucin and DDAB tertiary amino group present on the nanoparticles surface. Therefore, the reduction of ZP values observed for all NLC+ after 1 and 24 h of incubation with mucin could be attributed to the ionic interaction between the negatively charged mucin particles and NLC+. As a result, the interaction led to a decrease in NLC+ motion, which in turn could decrease their wash out by nasal mucociliary motion after administration, limiting the loss due to sneezing, thus allowing the sustained and prolonged release of the EO to directly reach the brain. These findings are very promising, because, although nasal drug delivery offers direct access of the therapeutics to the brain, it faces hindrances, such as the mucociliary clearance, that prevents drug retention at the mucosal surface with consequent loss of drug therapeutic effectiveness [80,81]. Mucoadhesive carriers could overcome this issue by prolonging the residence time of the drug in the nasal area, thereby increasing absorption and resulting in a remarkable therapeutic response [81].

## 4. Conclusions

Taken together, our results show that the use of *Lavandula* or *Rosmarinus* allowed the formation of smaller and more homogeneous nanoparticles, with a more ordered structure related to the formation of oily droplets into the lipid matrix compared to the other tested oils. The results of in vitro studies show that EO nanoencapsulation provides a sustained and prolonged release. Furthermore, *Lavandula* and *Rosmarinus* NLC were safe on both murine and human cell lines, at all tested concentrations. This preliminary study suggests that optimized positively charged NLC containing *Lavandula* or *Rosmarinus* can be proposed as a potential add-on strategy in the treatment of neurodegenerative diseases through intranasal administration, due to the well-known beneficial effects of essential oils and the mucoadhesive properties of the prepared NLC.

## Figures and Tables

**Table 1:** Values of pH, osmolarity, and entrapment efficiency (EE%) ± standard deviation (SD) of NLC prepared with Lavandula (L-NLC), Mentha (M-NLC), Rosmarinus (R-NLC), Tegosoft CT (CT-NLC), and Neem (N-NLC).

Sample	pH ± SD	Osmolarity (mOsm/kg ± SD)	EE% ± SD
L-NLC	7.13 ± 0.5	299.5 ± 0.1	76.47 ± 0.8
M-NLC	7.19 ± 0.6	293.1 ± 0.3	67.15 ± 0.3
R-NLC	7.20 ± 0.5	291.0 ± 0.5	64.09 ± 0.9
CT-NLC	7.28 ± 0.7	297.9 ± 0.2	100.00 ± 0.0
N-NLC	7.09 ± 0.4	291.5 ± 0.7	19.61 ± 0.8

**Table 2:** Turbiscan Stability Index (TSI) of NLC analyzed for 30 days at 25 °C using Turbiscan^®^ Aging Station. * Significance for *p* < 0.05.

Sample	Day of the Measurement				
	Day 1	Day 7	Day 14	Day 21	Day 28
CT-NLC	0.42	2.28	6.03	8.60 *	11.16 *
L-NLC	0.38	1.73	2.39	3.14	5.99
R-NLC	0.37	1.74	2.33	3.02	5.96
M-NLC	0.40	1.62	3.98	5.28	7.02 *
N-NLC	0.40	1.61	3.86	5.15	6.52

**Table 3:** Common vibrational modes.

Functional Group Mode	Approximate Wave Number (cm^−1^)
-CH_3_, symmetric and antisymmetric stretch	2920–2960
-CH_3_, symmetric and antisymmetric stretch	2850–2890
-PO_2_, symmetric and antisymmetric stretch	1080–1200
-C-C-	1050–1150
-C-O-	1410
-C=O	1720
-COH	870–890
-OH	1080–1090
-CH_2_-, deformation	1460–1470

**Table 4:** Raman intensity ratios related to C-C stretching vibrational bands (I_1115_/I_1050_) and C-H stretching vibrational bands I_2890_/I_2850_.

Sample	I_1115_/I_1050_	I_2890_/I_2850_
CT-NLC	0.69	1.20
L-NLC	0.74	1.94
M-NLC	0.53	0.50
N-NLC	0.71	0.62
R-NLC	0.67	1.00

**Table 5:** Crystallinity (C%) of Tegosoft CT NLC (CT-NLC), Neem NLC (N-NLC), Mentha NLC (M-NLC), Lavandula NLC (L-NLC), and Rosmarinus NLC (R-NLC).

Sample	C (%)
CT-NLC	10%
N-NLC	15%
M-NLC	30%
L-NLC	68%
R-NLC	40%

## Data Availability

The data presented in this study are available on request from the corresponding author.

## Supplementary Materials

The following are available online at https://www.mdpi.com/article/10.3390/pharmaceutics13081166/s1, Figure S1: Mean particle size (Zave, nm), polydispersity index (PDI) and Zeta Potentia (ZP) ± standard deviation (SD) of the prepared Lavandula NLC (L-NLC), Mentha NLC (M-NLC), Rosmarinus NLC (R-NLC), Tegosoft CT NLC (CT-NLC) and Neem NLC (N-NLC) analysed after 30 days of storage in Turbiscan, Figure S2: Raman spectra of all the prepared NLC, Figure S3: XRD spectra of all the prepared NLC, Figure S4: Transmission electron microscopy (TEM) images of NLC prepared using Mentha EO (M-NLC), Figure S5: Transmission electron microscopy (TEM) images of NLC prepared using Rosmarinus EO (R-NLC), Table S1: Raman parameters for the C-H stretching vibrational bands of NLC systems.
