# Supplementary material for: Essential Oil-Loaded NLC for Potential Intranasal Administration
Source: Pharmaceutics. 2021 Jul 28;13(8):1166. doi: 10.3390/pharmaceutics13081166 (PMC8399280; doi:10.3390/pharmaceutics13081166)
Supplement: Supplementary file 1 [file pharmaceutics-13-01166-s001.zip › pharmaceutics-1261648-supplementary.pdf]

pharmaceutics

MDPI

Supplementary Materials: Essential Oil-Loaded NLC for Potential Intranasal Administration

Angela Bonaccorso, Cinzia Cimino, Daniela Erminia Manno, Barbara Tomasello, Antonio Serra, Teresa Musumeci, Giovanni Puglisi, Rosario Pignatello and Claudia Carbone

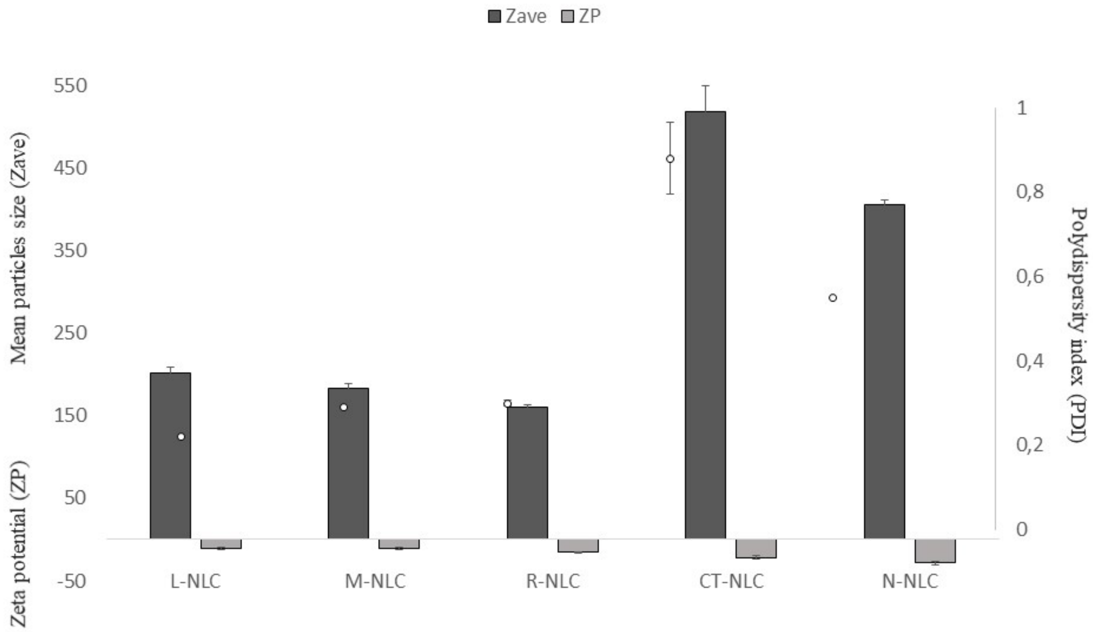

Figure S1. Mean particle size (Zave, nm), polydispersity index (PDI) and Zeta Potentia (ZP) ± standard deviation (SD) of the prepared Lavandula NLC (L-NLC), Mentha NLC (M-NLC), Rosmarinus NLC (R-NLC), Tegosoft CT NLC (CT-NLC) and Neem NLC (N-NLC) analysed after 30 days of storage in Turbiscan.

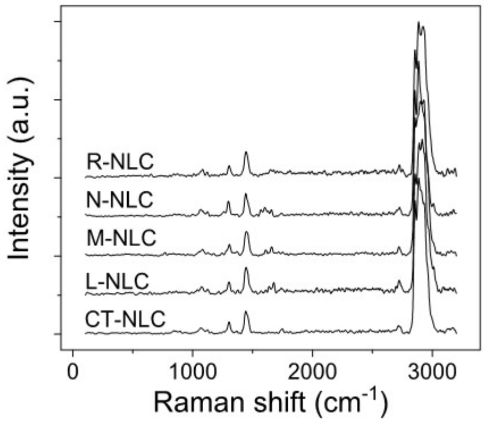

Figure S2. Raman spectra of all the prepared NLC.

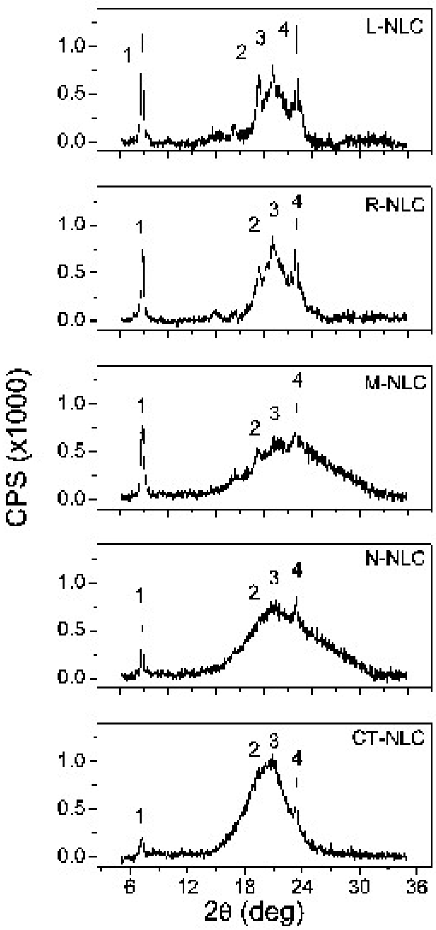

Figure S3. XRD spectra of all the prepared NLC.

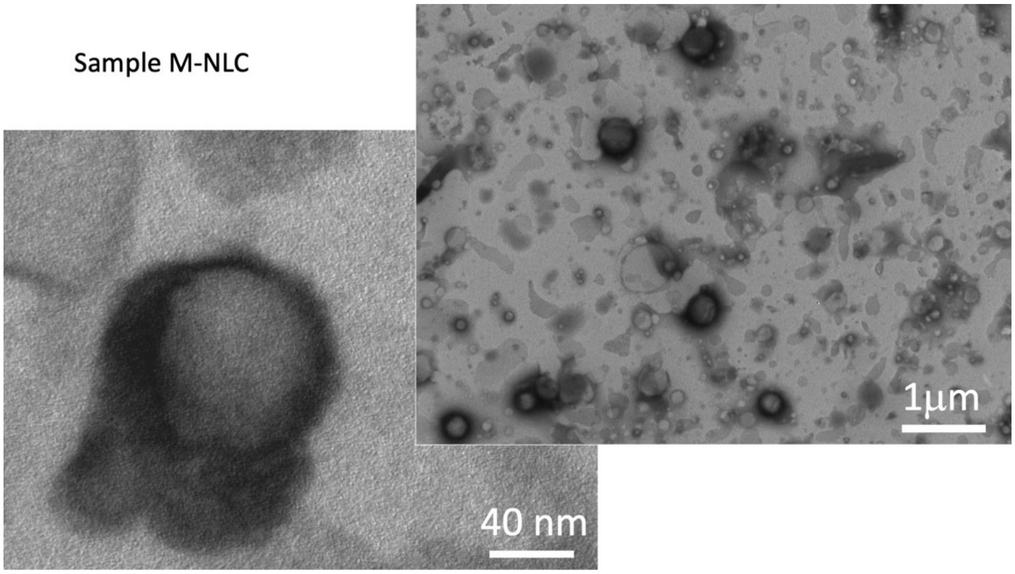

Figure S4. Transmission electron microscopy (TEM) images of NLC prepared using *Mentha* EO (M-NLC).

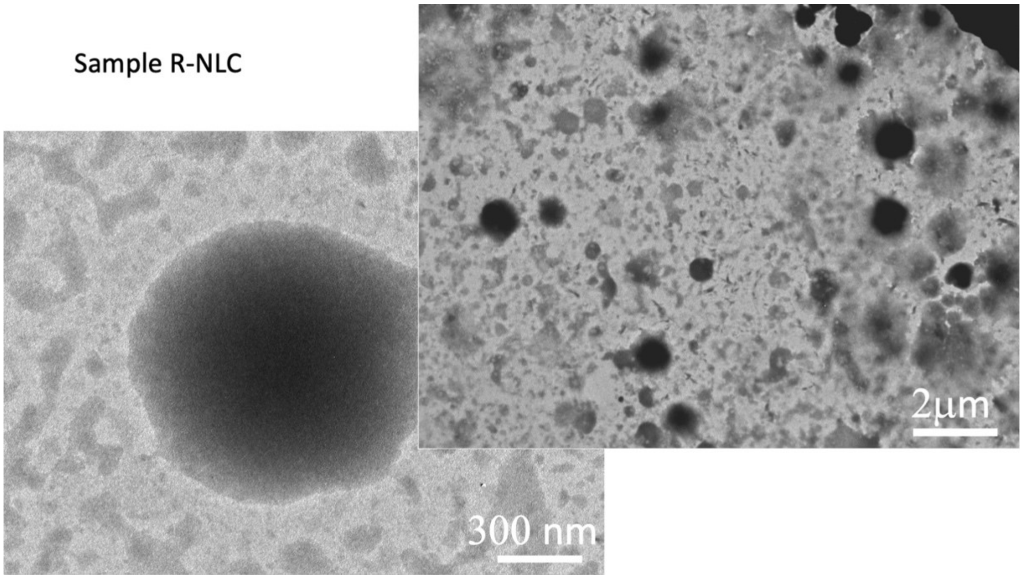

Figure S5. Transmission electron microscopy (TEM) images of NLC prepared using *Rosmarinus* EO (R-NLC).

Table S1. Raman parameters for the C-H stretching vibrational bands of NLC systems.

Sample	*peak position (cm^{-1})*	*FWHM (cm^{-1})*	*strength (a.u.)*
**CT-NLC**	2853	19	0.75
	2883	27	0.92
	2912	25	0.76
	2937	22	0.68
	2962	19	0.32
	2986	36	0.11
**L-NLC**	2853	16	0.49
	2890	47	0.95
	2916	16	0.29
	2936	25	0.71
	2969	27	0.38
	3007	32	0.17
**M-NLC**	2852	15	0.53
	2881	19	0.25
	2907	60	0.97
	2934	22	0.39
	2961	23	0.33
	2983	48	0.12
**N-NLC**	2853	18	0.64
	2882	18	0.40
	2902	58	0.76
	2936	19	0.22
	2963	24	0.20
	3001	34	0.06
**R-NLC**	2853	18	0.7
	2881	27	0.71
	2923	50	0.96
	2966	12	0.13
	2978	47	0.21
	3010	30	0.02
